# Its Origin, History, and Objects

**Published:** 1913-07-05

**Authors:** 


### THE NATIONAL MEDICAL GUILD.

### *Its Origin, History, and Objects.*

In the history of any organisation there comes a time when expansion is a necessary complement to existence. The growing body must expand and find fresh channels for its activity if it would avoid stagnation. So it is with the National Medical Guild; and these articles are written to introduce that lusty infant to a larger public than it has hitherto appealed to.

In the month of January of the present year an organisation known as the London Medical Committee was actively pursuing a campaign in London with the object of persuading the Insurance Committee to put into force the spirit of Clause 15 (3) of the National Insurance Act. To the original founders of that Committee were attracted a large band of most devoted workers whose efforts in the cause of the profession will be a matter for future historians of the fight to speak of. Suffice to say that it included in its ranks the majority of the best organising talent in London. Amongst these men the idea swiftly grew that the safety of the profession from future attacks rested in a strong combination that possessed powers which the existing medical bodies had never sought, and so a committee was deputed to make inquiry into the matter and report. From that inquiry and report sprang the National Medical Guild.

### The Growth of the Guild.

The Guild first came into existence on March 1, 1913, with offices in the neighbourhood of the Strand, a small membership list, and a Council filled with enthusiasm for the new project. That enthusiasm has more than justified itself. In the space of four months from a small body confined to the Metropolis the Guild has grown, and can now boast of branches in Kent, Sussex, Isle of Wight, Gloucestershire, Glamorgan, Monmouth, Essex, Worcestershire, Lancashire, the North of Scotland, and elsewhere. The fact that all these branches have been founded in response to inquiries from the districts concerned justifies the Council in appealing to a larger public.

### Some of its Chief Objects.

Under these circumstances it may not be out of place to give a précis of the objects of the Guild. The Guild stands for the protection of the profession as a whole, its ideal being a *free* profession doing its work unhampered by the red tape of excessive State or lay control. At the present moment most men's interests are centred in the Insurance Act, and its effect upon their professional and financial status; it is therefore necessary that the attitude of the Guild to this Act should be defined clearly.

This body recognises that there is a great difference between the practitioner who, in defiance of the British Medical Association and regardless of his own pledged word, joined the panel in the early days of January, and the later recruit who accepted service under the Act for economic reasons, and feels that the interests of the latter are identical with those of the men who still hold off. The policy of the Guild is opposed to the present administration of the Insurance Act; at the same time it is willing to afford the community every assistance in developing a scheme of Insurance in which the profession can co-operate willingly. If, therefore, it be granted that the interests of the non-panel and of the unwilling panel practitioner be identical, then it will readily be seen that the Guild can support the interests of both. In these aims there are involved no politics and no antagonism to other bodies in their work on behalf of the profession.

It is of paramount importance that any body that claims to be able to protect the interests of the profession should be in a position to take action both speedily and fearlessly and with the fullest immunity that the law allows. This can only be attained by embracing the privileges that are granted to Trade Unions. The experience of Germany and Australia incontestably proves the truth of this proposition, and however repugnant it may be to the professional mind, now that the State has interfered with the practice of more than three-quarters of the medical profession, a Trade Union affords the only means by which it can protect itself in the future.

The chief—indeed, the only—opposition that the Guild has had to encounter so far is the use of the term Trade Union; but it must be remembered that the work of Trade Unions does not consist entirely of strikes and peaceful picketing; these are, indeed, only adjuncts, which have been subjected to grave abuses in the past. The organisation that the Guild is anxious to build up may be compared to the position of a nation desirous of peace yet fully prepared for war. Had there been any such organisation in existence two years ago the position of the profession would have been unassailable.

Any unknown body starting out with high ideals such as those suggested in the foregoing must inevitably meet with opposition and difficulties, and it has been found, as a matter of experience, that the chief opposition arises from an imperfect acquaintance with the rules that form a Trade Union.

If asked to enumerate the chief stumbling-blocks over which intending members trip, one would put, in order of frequency, the question of levies, the attitude of the Guild to the Insurance Act, the legal position of a Trade Union, and the meaning of the Rules.

### The Question of Levies.

Firstly, as to levies. The largest sum that can be called up in any one year is £20, in four quarterly instalments. That is to say, the largest possible sum that can be levied at one time is £5, and three months' grace is allowed in which to pay it. But it is not to be assumed that such a sum would be wanted except under the gravest emergency, and it is extremely improbable that the full amount would ever be called up. Let it be assured that such an organisation has a membership of 25,000, and that during five peaceful years a call of £2 per member per annum was made. Apart from the accrued interest this would give a reserve fund of £250,000, and there would still remain the power of calling up the £20. Suppose the profession at this juncture were threatened by any grave encroachment on its interests, it would have behind it an actual fund of £250,000 and a potential £500,000 at call. If the critics of the late failure are correct in attributing it to lack of money, the profession would thus be in an unassailable position. Who would dare to attack a profession thus armed, or refuse to listen to its requests? Such a sum as £2 per annum is surely within the competence of any professional man, and the potential power of the £20 reserves would be a deterrent on anybody who sought to attack it.

The question of levies has been dealt with at some length because the organisers have found that some quite natural misgivings are aroused by the rule which empowers the Council to raise so large a sum in one call. It cannot be too strongly insisted that the power is one that will be allowed to remain dormant save in the gravest emergency.

### The Guild's Attitude to National Insurance.

The next objection to arise is the attitude of the Guild to the National Insurance Act, and here the position differs somewhat in London and the provinces. In London the majority of medical men are not on the panel, and they not unnaturally ask that the Guild shall be a non-panel body. In the country, on the contrary, the majority are working the Act, and look askance at any body that is appealing to non-panel men. The reconciliation of these apparently opposing interests is not so difficult as it would at first sight appear, and if it be agreed that the majority of the profession are giving unwilling service, and further that the interests of the unwilling servant and the non-panel practitioner are identical, then the Guild can appeal to both sections and can assist both parties in their desire to improve the service so that it may be worked by an honourable and a free profession.

### A Trade Union and the Guild's Rules.

In the next article the legal immunities and privileges of Trade Unions will be dealt with, and a brief *résumé* of the Rules of the Guild given.

